# Natural Phenolic Acid, Product of the Honey Bee, for the Control of Oxidative Stress, Peritoneal Angiogenesis, and Tumor Growth in Mice

**DOI:** 10.3390/molecules25235583

**Published:** 2020-11-27

**Authors:** Nada Oršolić, Martina Kunštić, Marina Kukolj, Dyana Odeh, Daniela Ančić

**Affiliations:** 1 Division of Animal Physiology, Faculty of Science, University of Zagreb, Rooseveltov trg 6, HR-10000 Zagreb, Croatia; martina.kunstic@gmail.com (M.K.); kukoljmarina@gmail.com (M.K.); dyana.odeh@biol.pmf.hr (D.O.); 2 Agency for Medicinal Products and Medical Devices, Ksaverska cesta 4, HR-10000 Zagreb, Croatia; daniela.vukasovic@gmail.com

**Keywords:** gallic acid, caffeic acid, Ehrlich ascites tumor, angiogenesis, macrophage activity, proangiogenic factors, mice

## Abstract

Tumor-associated macrophages (TAM) are key regulators of the link between inflammation and cancer, and the interplay between TAM and tumor cells represents a promising target of future therapeutic approaches. We investigated the effect of gallic acid (GA) and caffeic acid (CA) as strong antioxidant and anti-inflammatory agents on tumor growth, angiogenesis, macrophage polarization, and oxidative stress on the angiogenic model caused by the intraperitoneal (*ip*) inoculation of Ehrlich ascites tumor (EAT) cells (2.5 × 10^6^) in Swiss albino mouse. Treatment with GA or CA at a dose of 40 mg/kg and 80 mg/kg *ip* was started in exponential tumor growth phase on days 5, 7, 9, and 11. On day 13, the ascites volume and the total number and differential count of the cells present in the peritoneal cavity, the functional activity of macrophages, and the antioxidant and anti-angiogenic parameters were determined. The results show that phenolic acids inhibit the processes of angiogenesis and tumor growth, leading to the increased survival of EAT-bearing mice, through the protection of the tumoricidal efficacy of M1 macrophages and inhibition of proangiogenic factors, particularly VEGF, metalloproteinases -2 and -9, and cyclooxygenase-2 activity.

## 1. Introduction

Angiogenesis is the process of the formation of new capillary blood vessels from pre-existing ones and is considered essential for tumor growth and metastasis [1]. A growing tumor needs an extensive network of capillaries to provide nutrients and oxygen. In this process, tumor-associated macrophages (TAMs) play an important role. TAMs are key regulators of the link between inflammation and cancer. TAMs with other immune cells that infiltrate tumors are involved in dynamic interaction with tumor cells, and this interaction leads to tumorigenesis, metastasis, and drug resistance [2,3]. Thus, the interplay between TAMs and neoplastic cells represents a promising target of future therapeutic approaches. By the production of numerous enzymes, including matrix metalloproteinases 2 (MMP-2) and MMP-9 and their activators, such as chemokines, TAMs regulate the digestion of the extracellular matrix and exert immunosuppressive functions through: a) the release of anti-inflammatory cytokines; b) the modulation of tumor microenvironment producing survival/growth factors (e.g., vascular endothelial growth factor, VEGF); c) the facilitation of tumor progression via proangiogenic factor release (e.g., MMP-2 and MMP-9); d) an increase in the arginase activity (Arg) and a decrease in the activity of inducible nitric oxide synthase (iNOS) [2,3]. The MMP family comprises zinc-dependent endopeptidases involved in the remodeling and maintenance of the extracellular environment of cells that degrade both matrix and non-matrix proteins under physiological and pathological conditions. Dysregulated MMP activity leads to pathological conditions such as arthritis, nephritis, encephalomyelitis, intracerebral hemorrhage, chronic ulcers, fibrosis, inflammation, cancer, and metastasis [4]. In addition to the destruction of the basement membrane, MMPs are involved in promoting angiogenesis through the release of growth factors, including vascular endothelial growth factor (VEGF), cytokines, and other biologically active molecules that contribute to carcinogenesis. Additionally, they may potentially act as promising targets for cancer therapy. The tumor microenvironment continuously recruits inflammatory cells that produce large amounts of reactive oxygen species (ROS) that are important in all stages of tumor progression, from initiation to vascularization and metastasis [2,3,4,5]. ROS promote angiogenesis, either directly or via the generation of active oxidation products, including peroxidized lipids. The proangiogenic function of oxidative stress has been confirmed by the application of natural antioxidants, including numerous polyphenolic/flavonoid constituents such as caffeic acid, gallic acid, quercetin, and other flavonoids. Based on the above, the administration of natural antioxidants may be important in the therapeutic strategy against the tumor [2,3,4,5].

Numerous plant-derived compounds, including paclitaxel, docetaxel, vinblastine, vincristine, topotecan, irinotecan, etoposide, etc., have been linked to the chemoprevention and treatment of cancer [3,6]. The discovery of a new plant-derived agent that targets inflammation, MMPs, angiogenesis, and VEGF may be a promising therapeutic targets for many cancers [3,7].

Gallic acid (3,4,5-trihydroxybenzoic acid, GA) and caffeic acid (CA) are endogenous plant polyphenols abundantly found in honey bee products, tea, grapes, berries, and other fruits as well as in wine [8]. It has been demonstrated that GA and CA have antioxidant, anti-inflammatory, anti-mutagenic, anti-carcinogenic, anti-neoplastic, and apoptotic activities [3,8,9]. Furthermore, GA and CA have also been shown to suppress cell viability, proliferation, invasion, and angiogenesis in many human and animal cancer cell lines and in animal models [3,10,11,12,13,14,15]. Thus, Liu et al. [16] have shown that GA selectively induces apoptosis in pancreatic cancer cells without affecting the survival of normal cells, whereas Ho and colleagues [17] have shown that GA inhibits gastric cancer cell growth by modulating the levels of MMP-2 and -9 and cytoskeletal reorganization. The inhibitory effect of GA and CA on cancer cell growth is mediated via the modulation of different pathways, including cell cycle, metastasis, angiogenesis, and apoptosis. However, detailed molecular studies in vivo have not been carried out to examine the role of GA and CA on the peritoneal angiogenesis of Ehrlich ascites tumor in exponential growth phase and their effect on macrophage polarization and proangiogenic factors, particularly VEGF, MMP-2, MMP-9, and cyclooxygenase-2 (COX-2).

It should be noted that, in the US, there are about 250,000 new cases of cancer per year originating from organs in the peritoneal cavity [18], especially tumors of the ovaries, pancreas, colorectal, stomach, and liver, but also including extra-abdominal tumors of the lymphoma, lung, and breast. The problem with the treatment of these tumors is the microscopic remnants of the tumor and the escape of tumor cells into the abdominal cavity during surgery and the formation of ascites, which is an indicator of advanced tumor stage and poor survival rate (only 11% longer than 6 months) [19]. In this study, we used Ehrlich ascites tumor (EAT), which is a very suitable model because it can grow in both forms, as a solid and as an ascitic tumor. It is a fast-growing, angiogenesis-dependent tumor leading to increased vascular permeability, cell migration, and progressive ascites fluid formation, and is a suitable model for investigating the possible antiangiogenic effect of natural components on tumor growth. Precisely, EAT cells grow in two phases: a proliferating phase, in which the number of cells increases exponentially (up to the 9th day), and a plateau phase followed by a resting phase, in which the number of cells remains nearly constant [20]. EAT is an interesting model with which to analyze the interplay between the immune system, angiogenesis, and tumor development. The differences in angiogenesis and stroma generation between solid and ascites tumors relate primarily to kinetics; in ascites tumors, stromal elements accumulate in two separate compartments, such as ascites fluid in the peritoneal cavity and vascularized connective tissue in the tissues lining the peritoneal cavity, while in solid tumors extravasated plasma exudate and newly formed connective tissue form a single stromal compartment [21].

Since some studies showed that ROS regulate angiogenesis and tumor growth through VEGF [2,3,4,5], our goal was to investigate: a) the role of GA and CA in the regulation of ROS production, inflammation, angiogenesis, and tumor growth; b) the functional interplay between the levels of VEGF, ascites formation, and levels of MMP-2 and MMP-9; c) the antioxidant capacity of GA and CA in the reduction in ROS production and their role in the M1/M2 dichotomy following macrophage polarization; d) the role of CA and GA in macrophage polarization due to cytokine production, NO, arginase activity, ROS production, and tumor angiogenesis.

## 2. Results

### 2.1. Antitumor Activity of Galic and Caffeic Acid

We assessed the in vivo antitumor effect of the GA or CA using EAT-bearing mice as an animal model. The change in body weight (%), total number of EAT cells in the peritoneal cavity, survival of tumor-bearing mice, and tumor inhibition rates are shown in Table 1 and Table 2 and in the Supplementary Files (Figure S1).

The change in body weight of mice during therapy with GA or CA is an indicator of the rate of tumor growth or the formation of ascites in individual groups, and is expressed as the percentage change in relative body weight to the baseline weight. After 5 days of injection of tumor cells, an exponential increase in tumor growth and animal weight in the control group was found (Supplementary File, Figure S1). This increase was accompanied by an increase in tumor mass for up to 14 days, when the animals were sacrificed; the change in weight in the control group was 25.44% compared to the initial mass, while in animals treated with GA and CA at a dose of 40 and 80 mg/kg, the % change in weight was 19.29%, 16.33%, 17.66%, and 14.97%, respectively (Table 1).

The treatment of EAT-bearing mice in the exponential growth phase with both doses of GA and CA showed a significantly (*p* ≤ 0.05; 0.001 and *p* ≤ 0.01; 0.05) reduced total number of cells in the abdominal cavity compared to the control group. The total number of cells in the control group was 891.78 ± 76.32 (×10^6^), whereas it was 224.72 ± 69.24 (×10^6^) and 118.42 ± 15.96 (×10^6^) after treatment with GA, or 198.94 ± 41.12 (×10^6^) and 174.57 ± 15.80(×10^6^) in the groups treated with CA (40 and 80 mg/kg), respectively. If calculated as the % inhibition of tumor growth in relation to the control, the growth inhibition for GA (40 mg/kg and 80 mg/kg) was 74.80% and 86.72%, while for CA it was 77.69% and 80.42%, respectively (Table 1).

In the animals treated with GA and CA in relation to the control, the percentage reduction in the ascites fluid volume was 29.32%, 35.64%, 40.85%, and 34.41%, respectively (Table 1).

**Figure 1:**
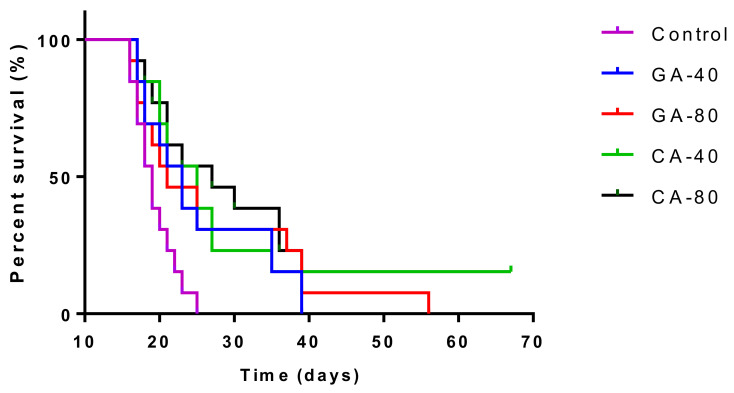
Kaplan–Meier survival curves of mice bearing EAT after treatment with GA and CA. Mice were injected intraperitoneally (*ip*) with 2.5 × 10^6^ viable EAT cells and treated with GA or CA at a dose of 40 and 80 mg/kg *ip* in exponential tumor ×growth phase on days 5, 7, 9, and 11. The results are expressed as the mean value of each experimental group ± SE of the mean (*n* = 11). Abbreviations: EAT, Ehrlich ascites tumor; GA, gallic acid; CA, caffeic acid.

The mean survival time of the control group was 19.29 ± 0.51 days, whereas it was 25.38 ± 0.94, 26.85 ± 3.23, 29.85 ± 4.97, and 32.38 ± 6.50 days for the groups treated with GA and CA (40 mg/kg and 80 mg/kg), respectively. The increase in the life span of tumor-bearing mice treated with GA and CA (40 mg/kg and 80 mg/kg) was found to be 31.57%, 39.19%, 54.74%, and 67.86%, respectively (Table 2). Kaplan–Meier survival curves of Swiss albino mice bearing EAT after treatment with GA and CA are presented in Figure 1.

### 2.2. Antiangiogenic Activity of Galic and Caffeic Acid

**Figure 2:**
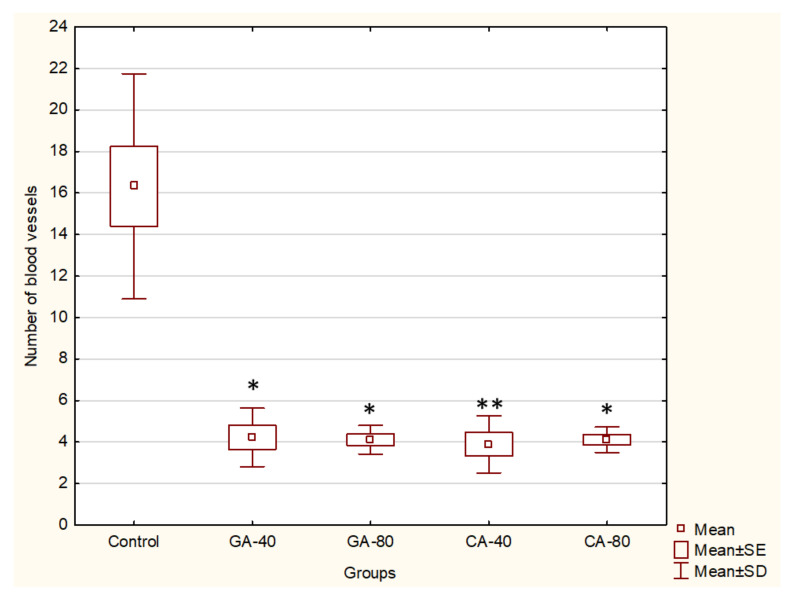
Effect of GA and CA on the microvessel density count (MVD) in the inner peritoneal lining of EAT tumor. Mice were injected intraperitoneally (*ip*) with 2.5 × 10^6^ viable EAT cells and treated with GA or CA at a dose of 40 mg/kg and 80 mg/kg *ip* in exponential tumor growth phase on days 5, 7, 9, and 11. Mice were sacrificed on the 13th day. The results are expressed as the mean value of each experimental group ± SE of the mean of three different observations; * *p* < 0.05, ** *p* < 0.01 as compared to the control group treated with saline. Abbreviations: EAT, Ehrlich ascites tumor; GA, gallic acid; CA, caffeic acid.

**Figure 3:**
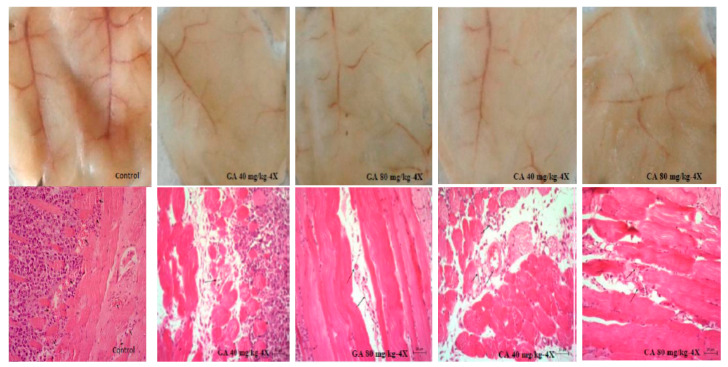
Effect of GA and CA on the suppression of angiogenesis in vivo. Inhibition of angiogenesis in GA or CA-treated mice is evident (above). Bouin-fixed, paraffin-embedded peritoneum of the control as well as the GA or CA-treated mice were sectioned (7 μm) and stained with hematoxylin and eosin (HE) dye and observed for microvessel density (MVD) at 200× magnification with a phase-contrast microscope. Decreased MVD in the peritonea of GA- or CA-treated mice is evident (below). Abbreviations: EAT, Ehrlich ascites tumor; GA, gallic acid; CA, caffeic acid.

Angiogenesis is required for multistage carcinogenesis process. Because GA and CA showed an inhibition of tumor growth and decreased ascites fluid volume, which is closely related to angiogenesis, we investigated the number of peritoneal vessels and the key factors in the process of angiogenesis, such as the VEGF and metalloproteinase activity. The results clearly indicated that there was a significant inhibition of neovascularization in mice treated with GA at both doses (40 mg/kg and 80 mg/kg, *p* ≤ 0.05) and CA (*p* ≤ 0.01 and *p* ≤ 0.05) as compared to the peritoneum of the control animals, which showed extensive neovasculature, as can be seen in Figure 2 and Figure 3.

When compared to 16.32 ± 1.91 microvessels present in the peritoneum of control EAT-bearing mice, the GA-treated mice peritonea showed 4.22 ± 0.57 and 4.11 ± 0.28 (at 40 or 80 mg/kg, respectively). The number of peritoneal blood vessels after the treatment of mice with CA was 3.88 ± 0.56 and 4.11 ± 0.25 (at 40 mg/kg or 80 mg/kg, respectively).

**Figure 4:**
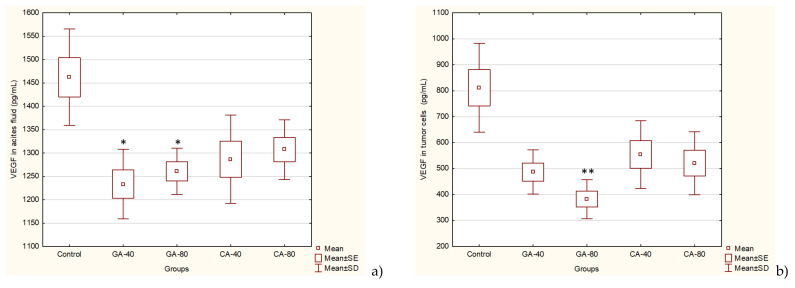
Effect of GA and CA on proangiogenic vascular endothelial growth factor (VEGF) in ascites fluid (**a**) and tumor cells (**b**) of mice bearing EAT. Mice were injected intraperitoneally (*ip*) with 2.5 × 10^6^ viable EAT cells and treated with GA or CA at a dose of 40 mg/kg and 80 mg/kg *ip* in exponential tumor growth phase on days 5, 7, 9, and 11. Mice were sacrificed on the 13th day, ascitic fluid and tumor cells were collected, and the VEGF levels were estimated by ELISA. The results are expressed as the mean value of each experimental group ± SE of the mean of three different observations; * *p* < 0.05, ** *p* < 0.01 as compared to the control group treated with saline. Abbreviations: EAT, Ehrlich ascites tumor; GA, gallic acid; CA, caffeic acid.

**Figure 5:**
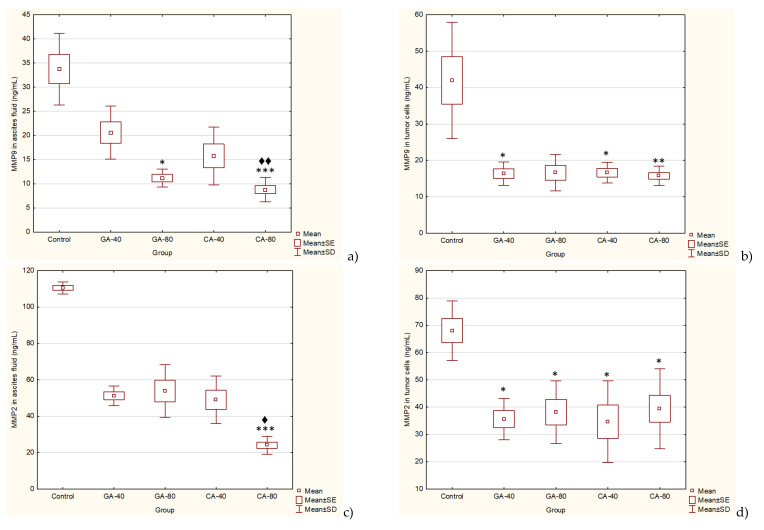
Effect of GA and CA on the MMP-9 (**a**, **b**) and MMP-2 (**c**, **d**) levels in ascites fluid and tumor cells of mice bearing EAT. Mice were injected intraperitoneally (*ip*) with 2.5 × 10^6^ viable EAT cells and treated with GA or CA at a dose of 40 and 80 mg/kg *ip* in exponential tumor growth phase on days 5, 7, 9, and 11. Mice were sacrificed on the 13th day, ascitic fluid and tumor cells were collected, and the MMP-9 or MMP-2 levels were estimated by ELISA. The results are expressed as the mean value of each experimental group ± SE of the mean of three different observations; * *p* < 0.05, ** p < 0.01, *** *p* < 0.001 as compared to the control group treated with saline; ^♦^ *p* < 0.05, ^♦♦^ *p* < 0.01 as compared to GA-40. Abbreviations: EAT, Ehrlich ascites tumor; GA, gallic acid; CA, caffeic acid.

EAT tumor is a rapidly growing tumor dependent on angiogenic factors, such as VEGF. The levels of angiogenic growth factor, VEGF, MMP2, and MMP9 secreted into ascites fluid during the growth of EAT cells and tumor cells were reduced in the presence GA or CA as compared to the saline-treated control group mice (Figure 4 and Figure 5).

GA successfully reduced the VEGF levels in tumor cells at a dose of 80 mg/kg (*p* ≤ 0.001) and in ascites fluid at both doses (*p* ≤ 0.05) as compared to the saline-treated control group, whereas CA reduced the VEGF levels but without statistical significance. However, a high percentage of reduction in the VEGF level was observed in tumor cells (~32% to 53%) in all experimental groups (Figure 4).

The results showed that GA reduced the level of MMP2 in the tumor cell lysate by 44.75% (GA, 40 mg/kg) or 48.46% (GA, 80 mg/kg), respectively, and in ascites fluid by 51.24% (GA, 40 mg/kg) or 53.70 (GA, 80 mg/kg) compared to the saline-treated control group. Additionally, the results showed that CA reduces the level of MMP2 in the tumor cell lysate by 42.89% (CA, 40 mg/kg) or 49.87% (CA, 80 mg/kg), respectively, and in ascites fluid by 55.62% (CA, 40 mg/kg) or 78.23% (CA, 80 mg/kg) compared to the saline-treated control group. The MMP9 levels were inhibited with GA and CA about 60% in tumor cells at both doses, while in ascites fluid GA inhibited MMP9 by 38.93% and 66.87% at dose of 40 or 80 mg/kg, while treatment with CA inhibited MMP9 by 53.28% and 73.94% at some doses compared to the saline-treated control group (Figure 5).

**Figure 6:**
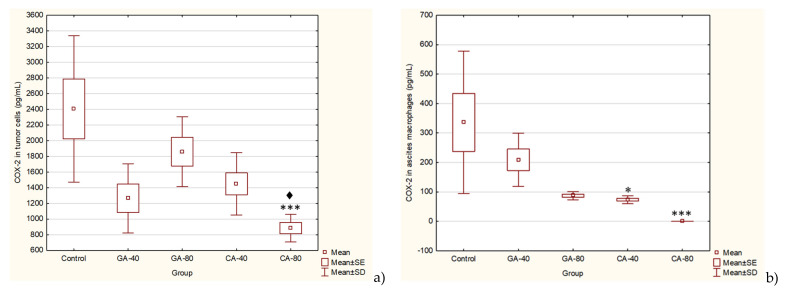
Effect of GA and CA on the COX-2 levels in tumor cells (**a**) and ascites macrophages (**b**) of mice bearing EAT. Mice were injected intraperitoneally (*ip*) with 2.5 × 10^6^ viable EAT cells and treated with GA or CA at a dose of 40 mg/kg and 80 mg/kg *ip* in exponential tumor growth phase on days 5, 7, 9, and 11. Mice were sacrificed on the 13th day, ascitic macrophages and tumor cells were collected, and the COX-2 levels were estimated by ELISA. The results are expressed as the mean value of each experimental group ± SE of the mean of three different observations; * *p* < 0.05, *** *p* < 0.001 as compared to the control group treated with saline; ^♦^ *p* < 0.05 as compared to GA-40. Abbreviations: EAT, Ehrlich ascites tumor; GA, gallic acid; CA, caffeic acid.

The inducible enzyme COX-2 is an important mediator of angiogenesis and tumor growth. It is known that inhibitors of COX-2 activity could be used as antiangiogenic agents for cancer prevention and treatment. CA significantly inhibited the level of COX-2 activity at a dose of 40 mg/kg (*p* ≤ 0.05) and completely (100%) in ascites macrophages and in tumor cells at dose of 80 mg/kg (*p* ≤ 0.001). Regardless of the reduced COX-2 level with GA in ascites macrophages (37.76 or 74.14%) and in tumor cells (47.42 or 22.72%), there was no statistical significance (Figure 6).

### 2.3. Effect of Caffeic Acid and Gallic Acid on Nitric Oxide and Arginase Activity

During the development of cancer, metabolic changes occur, including alterations in the enzyme activity of arginase and nitric oxide (NO) synthase (NOS), two enzymes that are important in tumorigenesis, especially in cancer cell proliferation, metastasis, tumor angiogenesis, and immunosuppression. Given the importance in the inhibition of polyamines, the product of arginase activity (Arg), and NO, we analyzed the level of Arg activity and NO in the supernatant of the spleen, ascites macrophages, and tumor cells.

Interestingly, the level of NO in the supernatant of the spleen macrophage was reduced in the CA-treated group as compared to the saline-treated control group (*p* ≤ 0.05), while the NO level in the supernatant of spleen macrophages in the GA-treated group did not change. The level of NO in the supernatant of the ascites macrophages in the GA-treated groups at doses of 40 mg/kg and 80 mg/kg was statistically higher as compared to the saline-treated control group (*p* ≤ 0.05 and *p* ≤ 0.01, respectively). The NO level was decreased in the ascites fluid in all treated groups, but it was statistically significant only at GA 40 mg/kg (*p* ≤ 0.001) and CA 40 mg/kg (*p* ≤ 0.05) as compared to the saline-treated control group (Table 3).

Previous reports have suggested that the arginase activity in cancer patients may arise from the tumor cells to sustain their rapid proliferation [22] or from the tumor microenvironment by mature myeloid cells [23].

The Arg1 levels were decreased in all treated groups; a statistically significant change was observed in the supernatant of splenic macrophages for GA at 40 mg/kg and 80 mg/kg (*p* ≤ 0.05, *p* ≤ 0.01, respectively) or CA at 80 mg/kg (*p* ≤ 0.01); in the supernatant of ascites macrophages for GA 80 mg/kg (*p* ≤ 0.05) and CA at a dose of 40 mg/kg (*p* ≤ 0.01) and 80 mg/kg (*p* ≤ 0.001); as well as in ascites fluid for GA at 40 mg/kg (*p* ≤ 0.001), GA at 80 mg/kg (*p* ≤ 0.05), and CA at 80 mg/kg (*p* ≤ 0.05) as compared to the saline-treated control group (Table 3).

### 2.4. Cytokine Levels in Ascites Fluid of EAT Bearing Mice

**Figure 7:**
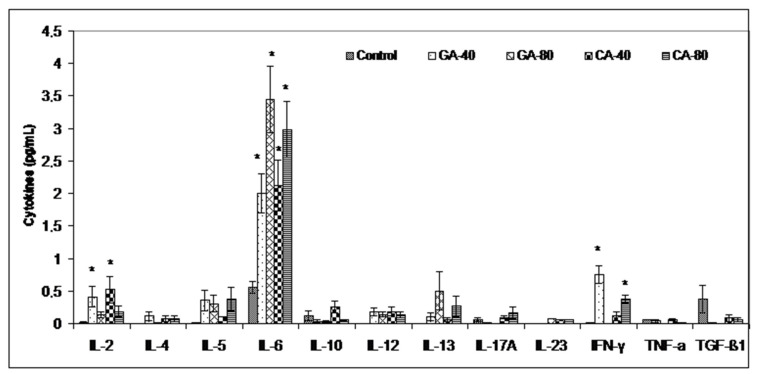
Effect of GA and CA on Th1, Th2, and Th17 cytokines in the ascites fluid of mice bearing EAT. Mice were injected intraperitoneally (*ip*) with 2.5 × 10^6^ viable EAT cells and treated with GA or CA at a dose of 40 mg/kg and 80 mg/kg *ip* in exponential tumor growth phase on days 5, 7, 9, and 11. Mice were sacrificed on the 13th day, ascitic fluid was collected, and cytokine levels were estimated by ELISA. The results are expressed as the mean value of each experimental group ± SE of the mean of three different observations; * *p* < 0.05 as compared to the control group treated with saline. Abbreviations: EAT, Ehrlich ascites tumor; GA, gallic acid; CA, caffeic acid.

The increased polyamine uptake, the product of arginase activity, by immune cells results in a lower production of tumoricidal cytokines by reducing the cytotoxicity of immune cells to the tumor. Thus, inhibiting the arginase activity by GA and CA in the tumor cell microenvironment may reverse the suppression of NO-mediated tumor cytotoxicity and may increase the tumoricidal activity of immune cells by increasing the Th1 cytokine activities and inhibiting tumor cell escape. Figure 7 clearly shows that the level of inflammatory Th1 cytokines was increased (IL-2, IL-6, IFN-γ) in relation to the saline-treated control group, indicating a possible polarization of M1 macrophages and an immunomodulatory effect of GA or CA.

### 2.5. Effect of Caffeic Acid and Gallic Acid on Oxidative Stress Markers

The presence of the tumor, the secretion of its toxic products, and the creation of inflammation and oxidative stress impair the function of all organs, including the liver and kidneys. The analysis of parameters such as oxidative stress and biochemical and hematological parameters (not presented here) may be an indicator of impaired renal function, while the use of natural antioxidants may reduce the resulting damage and improve renal function. As expected, GA or CA did not significantly alter markers of oxidative stress in the liver, kidney, and spleen, except for a slight reduction in malondialdehyde (MDA) and a slight increase in antioxidant capacity compared to the saline-treated control group (data not shown). However, large statistical changes in ascites fluid were also not observed, except for an increase in CAT activity in the GA (40 mg/kg) -treated group and a twofold increase in the superoxide dismutase (SOD) activity at both doses of GA compared to the saline-treated control group. A significant percentage reduction in the MDA levels (Table 4) was observed compared to the saline-treated control group as follows: CA-40 (52.02%), GA-40 (34.04%), GA-80 (25.26%), and CA-80 (10.56%). MDA reduction was followed by increased glutathione (GSH) levels (Table 4). High doses of GA and CA showed a decreased GSH level in comparation to the saline-treated control group, but without statistical significance.

**Figure 8:**
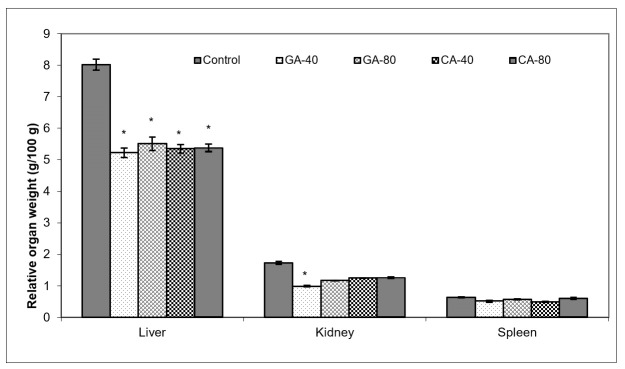
Effect of GA and CA on the relative organ weight of mice bearing EAT. Mice were injected intraperitoneally (*ip*) with 2.5 × 10^6^ viable EAT cells and treated with GA or CA at a dose of 40 mg/kg and 80 mg/kg *ip* in exponential tumor growth phase on days 5, 7, 9, and 11. The results are expressed as the mean value of each experimental group ± SE (*n* = 7). Mice were sacrificed on the 13th day; liver, kidney and spleen were collected immediately, weighed individually, and the relative weight was calculated as follows: relative organ weight (g/100 g) = total organ weight x 100/final body weight. Data are reported as the mean ± SE of the mean of three different observations; * *p* < 0.05, as compared to the control group treated with saline. Abbreviations: EAT, Ehrlich ascites tumor; GA, gallic acid; CA, caffeic acid.

Ascites is a prognostic sign of advanced cancer stage; the inflammatory infiltrate cells and cytokines in ascites may affect many functions of the vital organs, especially the liver, kidney, and spleen, even when the site of the tumor does not interfere directly with organ function. As seen in Figure 8, GA and CA reduced the relative organ weight, indicating the antioxidant and anti-inflammatory activity of the test components; an increase in the liver and kidney weight in the saline-treated control group may be an adaptive response to toxins from tumor cells accompanied by enhanced enzyme synthesis as a consequence of inflammatory cell infiltrate accumulation. GA-40 treated mice showed the best reduction in the relative weight of the liver and kidney (Figure 8).

## 3. Discussion

Given the complexity of tumor angiogenesis, pharmacological intervention is based on the modification of the structural and functional microenvironment of the tumor [24,25], especially by acting on factors that contribute to tumor progression, the long-term dormancy of tumor cells, and their survival.

In order to find a potent antiangiogenic, anti-inflammatory, and antioxidant drug, we investigated the effect of CA and GA on growth and angiogenesis in the exponential tumor growth phase of Ehrlich ascites tumor-bearing mice; *ip* EAT cell inoculation induces a strong local inflammatory response with increased vascular permeability, leading to an intense edema formation, cell migration, rapid proliferation, and an increase in ascites fluid formation (Table 1, Supplementary File, Figure S1). EAT-bearing mice treated with CA and GA showed a drastic reduction in tumor burden, EAT cell number, ascites formation, body weight, and secreted VEGF levels (Table 1, Figure 4 and Supplementary File, Figure S1).

The treatment of EAT-bearing mice in the exponential growth phase with both doses of GA and CA showed a strong effect on the reduction in the total number of cells in the abdominal cavity compared to the control group; the growth inhibition for GA (40 mg/kg and 80 mg/kg) was 74.80% and 86.72%, while for CA it was 77.69% and 80.42%, respectively. The percentage reduction in the ascites fluid volume in the animals treated with GA and CA compared to the control was 29.32%, 35.64%, 40.85%, and 34.41%, respectively. The levels of angiogenic growth factor, VEGF, MMP-2, and MMP-9 secreted into ascites fluid during the growth of EAT cells and tumor cells in the presence of GA or CA were reduced as compared to the control mice, while the life span of tumor-bearing mice treated with GA and CA (40 mg/kg and 80 mg/kg) was increased by 31.57%, 39.19%, 54.74%, and 67.86%, respectively (Table 2, Figure 1, Figure 4, and Figure 5).

It is known that increased NO and ROS generation in cancer cells may contribute to tumor angiogenesis [26] by upregulating VEGF and VEGF-induced neovascularization, which may increase the tumors’ metastatic ability. Besides VEGF, MMPs are known to be a part of different important pathways, including angiogenesis and metastatic process, oxidative stress, inflammation and fibrosis [27,28]. Inhibition of VEGF after CA or GA treatment led to the inhibition of ascites formation as well as a concomitant reduction in the peritoneal neovasculature and corresponding decrease in the MVD in the peritoneum of the treated mice (Figure 2, Figure 3 and Figure 4, Table 1). According to our data, it is possible that the reduction in oxidative stress in ascites fluid and the reduced NO and COX-2 levels contributed to direct inhibition of VEGF induction and the inhibition of M1 macrophage polarization in the M2 phenotype (Figure 6, Table 3). These processes may lead to the inhibition of cell proliferation, migration, and angiogenesis (Table 1, Figure 2 and Figure 3). Thus, ROS scavenging by antioxidants such as GA and CA could be used as antiangiogenic and anticancer therapeutics (Figure 6, Table 4).

VEGF is an endothelial cell-specific mitogen that plays a fundamental role in the fluid accumulation that is rich in nutritional factors for tumor growth [1,2,6]. Our data showed the significant bioactivity of CA and GA in the inhibition of tumor cell proliferation, tumor growth, and angiogenesis, indicating that CA and GA play a critical role in the inhibition of VEGF, MMP-2, and MMP-9 (Figure 4 and Figure 5); macrophage polarization (Table 3); cell migration; and the inhibition of ROS (Figure 6, Table 4); our findings are in accordance with the data of other authors [29]. Thus, through multiple mechanisms, including antitumor, antioxidant, anti-inflammatory, and immunomodulatory effects, GA or CA alter the tumor microenvironment, leading to a reduction in the VEGF levels and metalloproteinase activity; this results in a reduction in vascular density and permeability, and a reduction in the level of ascites fluid accumulation that is a crucial nutritional factor for the growth and survival of tumor cells.

Furthermore, the link between chronic inflammation and cancer involves cytokines and mediators of inflammatory pathways, such as COX-2. COX-2 is induced by pro-inflammatory cytokines at the site of inflammation, and the enhanced COX-2-induced synthesis of prostaglandins stimulates cancer cell proliferation, promotes angiogenesis, inhibits apoptosis, and increases metastatic potential [30]. CA significantly inhibited the level of COX-2 activity in ascites macrophages and in tumor cells at a dose of 80 mg/kg. CA and GA showed the dose-dependent inhibition of COX-2 activity in ascites macrophages (CA by 70% or 100% and GA by 37.76% or 74.14%) and in tumor cells (CA by 39.76 or 63.22 and GA by 22.72 or 47.42). According to these data, CA and GA are COX-2 inhibitors and could be explored for applications not only in the prevention and treatment of cancer but also in the inhibition of angiogenesis [31,32].

According to Harris et al. [30], by inhibiting the COX-2 activity with CA and GA, we not only inhibit ROS levels, inflammation, and carcinogenesis but also abolish the inhibition of B- and T- cell proliferation, particularly natural killer T cells, and enhance the antineoplastic activity of immune system cells. The inhibition of arginase activity also contributes to the strengthening of the immune response to tumor cells. Previous data suggested that arginase activity in cancer patients might come from the tumor cells and the tumor microenvironment, especially mature myeloid cells, which increase their rapid proliferation [22,23]. Thus, the inhibition of arginase activity in the tumor cell microenvironment may increase immune cell activity in killing tumor cells. Our data (Table 3) showed that the arginase activity is significantly higher in the ascites fluid of the control group compared to the GA- or CA-treated groups, while the NO levels in ascites fluid were decreased, especially in GA-40 and CA-40. GA-80 reduced the level of NO in the ascites fluid compared to the control, but without statistically significant differences. One possible explanation is the higher macrophage activity and higher NO production, while another explanation could be based on rapid reactivity NO with other radicals, including superoxide anion and peroxynitrite formation, which can be highly toxic to tumor cells [33]. Thus, a greater toxic effect of GA-80 on tumor cells may be mediated by peroxynitrites and their ability to inactivate mitochondria in tumor cells. Furthermore, the inhibition of arginase activity by GA and CA in the tumor cell microenvironment may reverse the suppression of NO-mediated tumor cytotoxicity and may increase the tumoricidal activity of macrophages and other immune cells by increasing the Th1 cytokine (IL-2, IL-6, IFN-γ) activities and inhibiting tumor cell escape (Figure 7). It has been shown that an increase in cytokines, such as IL-2 and IFN-γ, can activate NK cells, whose activity is reduced during EAT growth, and thus contribute to the natural resistance of tumor growth [3,34]. However, according to Spranger et al. [35] and Xu et al. [36], IFN-γ acts as an important cytokine in the tumor microenvironment that can potently induce the expression of PD-L1 and indoleamine-2,3-dioxygenase (IDO) in cancer cells to contribute to tumor immune evasion. It seems that GA-80, similar to apigenin, resveratrol, and curcumin, suppresses the IFN-γ-induced upregulation of PD-L1 and the IFN-induced activity of indoleamine-2,3-dioxygenase (IDO) via the inhibition of STAT1 phosphorylation, thus providing a reactivation of T cell functions with a concomitant increase in IL-2 synthesis and secretion [36,37]. On the other hand, it is known that cytokines are characterized by the property of pleotropism and redundancy and that can have completely different functions in the tumor microenvironment. Similar to cytokines, some polyphenols can act differently on cytokine production depending on the dose as well as tumor cells [38]. These data suggest immunomodulatory activity of CA and GA and their effect in an increase in the M1 tumoricidal efficacy of TAM and the blocking of the M2 tumor activity of TAM, which prevents the immune surveillance system by producing immuno-suppressing cytokines [39]. Interferon gamma is an immunoregulatory cytokine that also activates M1 macrophages as antigen presenting cells (APCs) with high levels of major histocompatibility complex (MHC) I and class II antigens and secret complement factors that facilitate complement-mediated phagocytosis [3,40]. M1 macrophages are important APCs, which can contribute to innate and adaptive immunity employing various cytokines and chemokines that can contribute in tumor destruction [3,39,40]. Ascites macrophages exhibit a different behavior in NO production between GA- and CA-treated mice. GA showed a better effect on the preservation of M1 ascites macrophages and NO production as opposed to CA; GA at both doses (40 mg/kg and 80 mg/kg) showed increased dose-dependent NO levels, while the arginase levels were significantly reduced only at higher doses. CA showed a significant dose-dependent decrease in Arg activity in ascites macrophages at both doses, while there was no difference between CA-treated mice and the control in terms of NO level. It seems that the preservation of M1 macrophages together with the inhibition of COX-2 activity could be a key mechanism in the inhibition of angiogenesis and tumor growth. Thus, CA appears to inhibit angiogenesis and tumor growth by inhibiting COX-2 activity as opposed to GA, whose mechanism is based on increasing the M1 TAM activity and the NO production in ascites macrophages.

Tumors in experimental animals or in human body are known to affect many functions of the vital organs. Ascites is a prognostic sign of advanced cancer stage and their inflammatory infiltrate cells and cytokines may affect many functions of the vital organs, especially the liver, kidney, and spleen, even when the site of the tumor does not interfere directly with organ function [13,41,42]. EAT cell inoculation into the mice causes changes in the balance of the antioxidant/oxidative stress system both in EAT and liver tissue [42]. ROS and lipid peroxidation play a role in cancer development, and many reports indicate that cancer cells require certain amounts of ROS for proliferation and survival. It is known that oxidative stress and levels of MDA, the end product of lipid peroxidation, were higher in carcinomatous tissue than in normal organs [3,43]. On the contrary, GSH, a potent inhibitor of the neoplastic process and plays an important role as an endogenous antioxidant system that is found particularly in high concentrations in liver and is known to have a key function in the protective process. Furthermore, the free radical scavenging system, SOD, and catalase are present in all oxygen metabolizing cells, and their function is to provide a defense against the potentially damaging reactivities of superoxide and hydrogen peroxide [3,42,43]. The reduction in oxidative stress and increase in antioxidant capacity led to a subsequent decrease in the injury and damage of the hepatocytes, kidney, or spleen membranes and the better functional activity of these organs in CA- or GA-treated EAT-tumor bearing mice. These changes were also visible in the reduction in the relative weight of the liver, and kidney, especially in GA-40 treated mice. The GA-40-treated mice showed a reduction in MDA and an increase in SOD and CAT activity in ascites fluid (Figure 8, Table 4). The group treated with CA-40 showed a significant reduction in the MDA levels in ascites fluid when compared with the control EAT-tumor bearing mice. The increase in MDA and decrease in SOD activity in the control EAT-bearing mice might be due to the loss of Mn SOD activity in EAT cells and mitochondrial dysfunction, leading to a decrease in the total SOD activity in the liver. A reduction in the SOD and CAT activities in the ascites fluid of control EAT-bearing mice may be the reason for tumor growth, while the treatment of mice with CA or GA controls the redox status of EAT-tumor bearing animals, thus increasing their survival (Table 2 and Table 4, Figure 1).

## 4. Materials and Methods

### 4.1. Animals and Ethics Statement

All the animal procedures were reviewed and approved by the ethical committee (Faculty of Science, University of Zagreb, Croatia) (approval code: 251-58-10617-16-1). Animal studies were performed in compliance with the guidelines in force in the Republic of Croatia (Law on the Welfare of Animals, N.N. #135, 2006; Regulations for the Environmental Conditions of Experimental Animals, Special Conditions for the Facilities and Experiment Categories, N.N. #176, 2004), and the Directive of The European Parliament and of the Council (2010/63/EU) and according to the Guide for the Care and Use of Laboratory Animals, DHHS Publ. (NIH) 86-123, 1985. Male Swiss albino inbred mice, weighing 20–25 g, approximately 2 months old, obtained from the Department of Animal Physiology, Faculty of Science, University of Zagreb, were used in this study. The animals were kept under a 12L:12D h light-dark regime at 60% humidity and were maintained on a pellet diet (Standard Diet 4RF 21 GLP certificate, Mucedola, Italy) and water ad libitum.

### 4.2. Tumor Cells

EAT is transplantable poorly differentiated and fast-growing malignant tumor which appeared originally as a spontaneous breast carcinoma in a mouse. EAT cells grow as an ascites tumor in the peritoneal cavity in Swiss albino mice. EAT was maintained by the intraperitoneal transplantation of EAT tumor cells into another mouse every 7 to 9 days. The viability of the prepared cells was greater than 95% (Trypan Blue test).

### 4.3. Phenolic Acid

Caffeic acid (CA)–3,4-di-hydroxycinnamic acid, (C_15_H_10_O_4_; Mw:180,2; purity ≥ 98%) was purchased from Sigma-Aldrich, St. Louis, MO, USA. Gallic acid, also known as 3,4,5-trihydroxybenzoic acid (C_7_H_6_O_5;_ Mw: 170,12; purity ≥ 98%), was purchased from Sigma-Aldrich, St. Louis, MO, USA. Test components were freshly prepared, dissolved in 0.9% saline, and given to mice intraperitoneally (*ip*) at a dose of 40 mg/kg and 80 mg/kg body weight [3,17].

### 4.4. Ehrlich Ascites Tumor (EAT) Model and Experimental Design

A total of 90 mice were used in this study. Ehrlich ascites tumor (EAT) is a suitable transplantable tumor model in order to study the antitumor, angiogenic, and antiinflammatory effects of the chemical and natural components. EAT cells were grown in the peritoneal cavity by the peritoneal inoculation of 0.5 mL of EAT viable cell suspension (2.5 × 10^6^) in 0.9% saline solution. The day of tumor inoculation was assigned as day “0”. On day 1, the animals were randomized and divided into five groups (*n* = 18 in each group)—a control and four experimental groups. Animals were treated *ip* with 0.9% saline solution (control EAT group) and with CA or GA at a dose of 40 mg/kg and 80 mg/kg/d/bw into the EAT bearing mice every alternate day after 5 days of tumor inoculation when tumor was in exponential growth phase on days 5, 7, 9, and 11. The weight of the animals was recorded ~ every five days from the day of inoculation to sacrifice.

Eleven animals from each group were left for monitoring the survival of animals and seven animals per group were sacrificed on the 13th day by an *ip* overdose of anesthetic Narketan^®^10 (Vetoquinol, Lure, France) at a dose of 100 mg/kg and analgesic Xylapan^®^ (Vetoquinol Biowet, Gorzow, Poland) at a dose 5 mg/kg and cervical dislocation. After sacrifice, the animals were used for harvesting the EAT cells and the analysis of the total cell number in the abdominal cavity; the volume of ascitic fluid; the differential count of the cells present in the intraperitoneal fluid, weight of liver, spleen, kidney and for a macrophage spreading test. Macrophages isolated from the peritoneal cavity of total ascites and spleen macrophages were used for nitric oxide (NO) and arginase 1 activity assay, while ascites fluid obtained after the centrifugation of ascites cells from the peritoneal cavity was used for an analysis of VEGF secretion, metalloproteinases 2 and 9 (ELISA Kit, Chongqing Biospes Co., Ltd.) and Th1, Th2, Th17 cytokines (Multi-Analyte ELISArray kit, Qiagen). In addition to ascites fluid, VEGF was also measured in the tumor cells and macrophages isolated from the peritoneal cavity of total ascites.

Liver, spleen, kidney were collected to determine oxidative stress parameters such as catalase (CAT), malondialdehyde (MDA, end product of lipid peroxidation), total glutathione (GSH), and superoxide dismutase (SOD). The peritoneal wall was excised, fixed, and used for microvessel density (MVD) and histological analysis.

### 4.5. In Vivo Studies

After the injection of EAT cells (2.5 × 10^6^) into the peritoneal cavity and treatment with CA and GA, animals were weighed from the initial day of tumor inoculation (day 0) to the end of the experiment (day 13) ~ every fifth day; a change in tumor weight was an indicator of tumor growth rate monitoring. Percentage change in weight was calculated for individual animals as:

(1) $$\mathrm{Percentage}\;\mathrm{change}\;\mathrm{in}\;\mathrm{weight}=\frac{\left(\mathrm{final}\;\mathrm{weight}\;-\;\mathrm{initial}\;\mathrm{weight}\right)\times 100}{\mathrm{final}\;\mathrm{weight}}$$

On the 13th day, 48 h after the last dose of CA or GA, each animal was weighed individually before blood was sampled. After sacrificing treated and control EAT bearing animals (*n* = 7), an incision was made in the abdominal region and *the* peritoneal fluid of all animals was collected and the volume, total number, and differential cell counts was determined in each animal. Total number of tumor cells present in the peritoneal fluid was counted with a hemocytometer using a dye-exclusion technique [3] and the percent of growth inhibition was calculated as follows:

(2) $$\mathrm{Tumor}\;\mathrm{inhibition}\;\left(\%\right)=\frac{\left(\mathrm{Av}.\;\mathrm{no}.\;\mathrm{of}\;\mathrm{cells}\;\mathrm{in}\;\mathrm{control}\;\mathrm{group}\;-\;\mathrm{av}.\;\mathrm{no}.\mathrm{of}\;\mathrm{cells}\;\mathrm{in}\;\exp.\;\mathrm{group}\right)\text{×}100}{\mathrm{Av}.\;\mathrm{no}.\;\mathrm{of}\;\mathrm{cells}\;\mathrm{in}\;\mathrm{control}\;\mathrm{group}}$$

Differential cell counts were determined by counting at least 800 cells after staining with May Grünwald in each sample and under microscope at 400x magnification and differentiated into macrophages, lymphocytes, and neutrophils, and tumor cells [3,40].

The liver, kidney and spleen samples were then obtained and weighed individually and the relative weight was calculated as:

(3) $$\mathrm{Relative}\;\mathrm{organ}\;\mathrm{weight}\;\left(\frac{g}{100}g\right)=\frac{\left(\mathrm{Total}\;\mathrm{organ}\;\mathrm{weight}\times 100\right)}{\mathrm{Final}\;\mathrm{body}\;\mathrm{weight}}$$

Survival analysis (*n* = 11 for groups) was evaluated by the surveillance of spontaneous death or by the selective killing of animals showing signs of pain or suffering according to established criteria described in paper [3]. Results were expressed as a percent of the mean survival time of the CA- or GA-treated animals over the mean survival time of the saline-treated control group (treated vs. control, T/C%) and as the percentage of increased lifespan (ILS%) by the formula:

(4) $$\mathrm{ILS}\%=\frac{\left(T\;-\;C\right)}{\;C\;}\times 100\;$$

where T represents the mean survival time of the treated animals and C the mean survival time of the control group.

### 4.6. Preparation of Macrophage from Peritoneal Cavity and Spleen and Evaluation of Its Functional Activity and Polarization

After collecting ascites from the abdominal cavity, macrophages were obtained by incubating 1 × 10^6^ cells/mL for 3 h in 24-well microplates in Dulbecco’s Modified Eagle’s Medium (DMEM medium) with 10% inactivated Fetal Calf Serum (FCS) at 37 °C in an atmosphere of 5% CO_2_, as described in the paper [3]. After the removal of non-adherent cells and rinsing with phosphate-buffered saline (PBS), macrophages were incubated for the next 24 h with or without 0.1 μg/mL lipopolysaccharide (LPS) and supernatant, and macrophages were used for NO and arginase 1 analysis.

Spleens were dissected from the abdominal cavity and the mononuclear cells were extracted using the “Lymphoprep” (Nyegard, Oslo, Norway) separation media. A mononuclear cell layer was collected, washed twice with medium, and adjusted to 1 × 10^6^ cells/mL with 0.1 μg/mL of LPS in DMEM containing 10% FCS at 37 °C and in atmosphere of 5% CO_2_.

NO was measured by the Griess reaction as the amount of produced NO_3_ and NO_2_, using a nitrate/nitrite assay kit (Griess Reagent System, Promega, USA). The absorbance at 540 nm was recorded using a microplate spectrophotometer, the Ao Absorbance Microplate Reader (Azure Biosystems, SAD). Optical density measurements were averaged and converted to µM nitrites using a standard curve of sodium nitrite. The limit of detection was 2.5 μM.

Arginase activity (ARG) was measured by Arginase activity assay kit MAK112 (Sigma-Aldrich, St. Louis, MO, USA.) in cell supernatant and cell lysates of adherent cells obtained by scraping the plastic surface with a rubber policeman as previously described [3] according to the manufacturer’s instructions. In this assay, arginase catalyzes the conversion of arginine to urea and ornithine and arginase activity was based on the ARG amount that converted L-arginine to 1.0 μmol of urea per min at 37 °C. Upon addition of the urea reagent, the urea reacts to form a colored product, proportional to arginase activity and urea concentration was determined spectrophotometrically at the absorbance 430 nm (Ao Absorbance Microplate Reader, Azure Biosystems, SAD).

The functional activity of macrophages in the peritoneal cavity was determined to the already described spreading technique [3,40], where 200 macrophages were scored as either round or spread and macrophage spreading (SI) index calculates as follows:

(5) $$\mathrm{SI}=\;\frac{\left(\mathrm{number}\;\mathrm{of}\;\mathrm{spreading}\;\mathrm{macrophages}\times 100\right)}{200\;\mathrm{adherent}\;\mathrm{cells}}$$

where SI = % of spreading macrophages. Wet adherent cells were photographed at 400× and 1000× magnifications with a phase-contrast microscope.

### 4.7. Quantification of VEGF, MMP2, MMP9, COX-2 and Cytokines Level in EAT Cells

After sacrificing mice, peritoneal lavage was collected and centrifuged at 10000 rpm for 10 min, and the ascites fluid was collected. Acites fluid and tumor homogenates or macrophage cells from peritoneal cavity were used for VEGF, MMP-2, MMP-9, COX-2, and cytokine quantification by ELISA according to the manufacturer’s instructions as already detailed [3]. The VEGF was measured by using the ELISA kit (Mouse VEGF Immunoassay, Quantikine^®^ ELISA, R&D Systems Europe, Ltd., UK); MMP-2 and MMP-9 concentrations were measured by Mouse MMP-2 and MMP-9 ELISA Kit (Chongqing Biospes Co., Ltd.); COX-2 by Human/Mouse Total COX-2 (R&D Systems Europe, Ltd., UK); and cytokines by Multi-Analyte ELISA Array kit (Qiagen).

VEGF measurement employed the quantitative sandwich enzyme immunoassay technique. A polyclonal antibody specific for VEGF has been pre-coated onto a microplate. Standards, control, and samples were pipetted into the wells and VEGF present was bound by the immobilized antibody. After washing the microplate, an enzyme-linked polyclonal antibody specific for mouse VEGF was added to the wells. After washing the microplate again, a substrate solution was added to the wells. The enzyme reaction gave a blue product which turned yellow by the addition of Stop Solution. Color measured is in proportion to the amount of VEGF bound in the initial step. The sample values were then read at 450 nm with the correction wavelength set at 540 nm (Ao Absorbance Microplate Reader, Azure Biosystems, SAD).

For the MMP-2 and MMP-9 measurements, 100 μL of standard or appropriate sample was added to a 96-well microtiter plate and incubated for 90 min at 37 °C to allow binding of MMP-2 or MMP-9 to the immobilized antibody. Unbound substrate was washed away, and an enzyme-linked polyclonal antibody specific for mouse MMP-2 or MMP-9 was added to the wells, followed by subsequent washes and the addition of color substrate. The absorbance values of the samples were read at 450 nm on Ao Absorbance Microplate Reader (Azure Biosystems, SAD), and the mean MMP-2 and MMP-9 protein levels from each triplicate were used for statistical analysis.

The method to measure COX-2 concentration is based on the sandwich ELISA technique, where immobilized binding antibodies specifically bind to COX-2. After washing the unbound material, a biotin-bound detection antibody specific for COX-2 is used to detect bound proteins using the streptavidin-HRP conjugate. Sample absorbance values were read at 450 nm on an Ao Absorbance Microplate Reader (Azure Biosystems, SAD), with a wavelength correction at 540 nm. As a standard, we used 120 ng/mL of total COX-2 standard from a kit freshly prepared with sample dilution buffer/standard in the concentration range of 0 to 10,000 pg/mL.

The cytokine Th1, Th2, and Th17 quantification in the unknown samples was determined from the absorbance read on an Ao Absorbance Microplate Reader (Azure Biosystems, SAD) at 450 and 570 nm. Multi-Analyte ELISA Array kit, Qiagen assay detects IL-2, IL-4, IL-5, IL-10, IL-12, IL-13, Il-17A, Il-23, IFN-γ and TNF-α, TGF-β1.

### 4.8. Peritoneal Angiogenesis and Microvessel Density (MVD)

After harvesting the EAT cells, the peritoneum was removed from EAT bearing mice, either treated or untreated mice. Samples were fixed in 10% neutral buffered Bouin fixative, washed, dehydrated and embedded in paraplast (24 h) for histological analysis. Paraffin embedded tissues of peritoneum were taken, and 7 µm sections were prepared and stained with HE. The blood vessels were counted under a light microscope at 400x magnification (Nikon Eclipse E600). MVD was determined in areas of invasive tumor containing the highest numbers of capillaries and microvessels per area. Individual microvessel counts were made on a 200x field within the area of most intense tumor neovascularization. Results were expressed as the highest number of microvessels identified within any single 200x field. Photomicrographs were taken using a digital camera (AxioCam ERc5s, Zeiss) and processed with computer program for morphometric image analysis (AxioCam ERc5s-ZEN2) [44].

### 4.9. Tissue Preparations for Quantitative Analysis of Oxidative Stress Biomarkers

Liver, kidney, spleen, and ascites fluid samples were prepared as described by Oršolić et al. [45,46]. Homogenates were centrifuged by Micro 200R centrifuge (Hettich) for 15 min at a speed of 10 000 rpm at +4 °C. The supernatant was used for the measurements of glutathione (GSH), the lipid peroxidation level (MDA), superoxide dismutase (SOD), and catalase (CAT) activity. All methods have been previously described [3,45,46], and all parameters were normalized in relation to exact protein content.

Briefly, the presence of lipid peroxidation was determined by measuring the concentration of malondialdehyde (MDA) that reacts with thiobarbituric acid and produces chromogen that is measured spectrophotometrically at 532 nm with a Libra S22 spectrophotometer (Biochrom, UK) as already described in [3,45,46].

The total GSH level was determined by 5-5’-dithiobis [2-nitrobenzoic acid] (DTNB, Ellman’s Reagent) and measured on the spectrophotometer at 412 nm, as described before [3,45].

The SOD activity was calculated from the percentage of inhibition of the reaction of xanthine oxidation by xanthine oxidase (optimized reaction ratio ΔA/min ≈ 0,025), which creates superoxide anion as a substrate for SOD present in samples. The percentage of inhibition was calculated using a calibration curve of different dilutions of SOD enzyme values, as already described in another article [3,45].

With the spectrophotometric method by Aebi at a wavelength of 240 nm, the catalase activity (CAT) was determined as the amount of hydrogen peroxide (H_2_O_2_) depleted over one minute. The concentration obtained is expressed as U/mg protein, which corresponds to μmol of degraded H_2_O_2_ per minute per milligram of protein [47].

### 4.10. Statistical Analysis

Statistical analyses were performed using the STATISTICA 13 software (StatSoft, Tulsa, OK, USA) and data were presented as mean ± standard error (SE) of the mean. By Kruscal–Wallis ANOVA test, all data were analyzed and further analyses of the differences between the groups were made with a multiple comparisons of mean ranks for all groups.

The data were considered significant at *p* < 0.05. Treatment-dose specific survival curves were calculated by the Kaplan–Meier method [48], and a comparison between the survival curves was made by log-rank test (α = 5 %) [49].

## 5. Conclusions

To summarize, on the basis of the results obtained herein and studies by other authors, we believe that CA and GA have a great potential against ascitic EAT tumor through a pleiotropic molecular mechanism(s) of action, including the inhibition of tumor cell proliferation, tumor growth, invasion, and angiogenesis. These effects may be primarily due to a specific effect on the inhibition of VEGF, MMP2, MMP9, ROS, and COX-2 activity and the maintenance of M1 macrophage activity. Antiangiogenic and antitumor activity together with the control of endogenous redox state and inflammation process by CA or GA may contribute to a reduction in the damage of vital organs, including liver, kidney, and spleen and improve their functional activity, along with increasing the lifespan of EAT tumor-bearing mice.

## Figures and Tables

**Table 1:** Effect of GA and CA on the total number of cells, tumor growth inhibition, ascites volume, and animal weight change (%) of mice bearing EAT.

Experimental Group ^a^	Total Number of Cells (×10^6^)	Min-Max Value (×10^6^)	Inhibition of Tumor Growth (%)	Volume of Ascitic Fluid (mL)	Min-Max Value	Inhibition of Ascitic Fluid%	% of Animal Weight Change
Control	891.78 ±76.32	672.00–1192.90	-	14.00 ± 0.43	12.10–15.60		25.44
GA 40 mg/kg	224.72 ± 69.24 *	219.60–554.40	74.80	9.88 ± 1.19 *	6.00–14.10	29.32	19.29
GA 80 mg/kg	118.42 ± 15.96 ***	77.35–200.81	86.72	9.00 ± 0.58 **	7.10–11.90	35.64	16.33
CA 40 mg/kg	198.94 ± 41.12 **	280.45–412.80	77.69	8.27 ± 0.39 ***	7.00–11.00	40.85	17.66
CA 80 mg/kg	174.57 ± 15.80 **	139.20–260.00	80.42	9.17 ± 0.57 *	7.20–11.60	34.41	14.97

^a^ Mice were injected intraperitoneally (*ip*) with 2.5 × 10^6^ viable EAT cells and treated with GA or CA at a dose of 40 mg/kg and 80 mg/kg *ip* in exponential tumor growth phase on days 5, 7, 9, and 11. The results are expressed as the mean value of each experimental group ± SE of the mean of three different observations; * *p* < 0.05;** *p* < 0.01; *** *p* < 0,001 as compared to the control group treated with saline. Abbreviations: EAT, Ehrlich ascites tumor; GA, gallic acid; CA, caffeic acid.

**Table 2:** Effect of GA and CA on the survival range, mean survival time, and increased life span (%) of mice bearing EAT.

Experimental Group ^a^	Survival Range (Days)	Mean Survival (Days)	% T/C ^b^	% ILS ^c^	LTS% ^d^
Control	16–25	19.29 ± 0.51	/	/	0
GA 40 mg/kg	17–25	25.38 ± 0.94	131.59	31.57	0
GA 80 mg/kg	14–56	26.85 ± 3.23	139.17	39.19	0
CA 40 mg/kg	14–67	29.85 ± 4.97	154.72	54.74	2
CA 80 mg/kg	17–67	32.38 ± 6.50	167.88	67.86	2

^a^ Mice were injected intraperitoneally (*ip*) with 2.5 × 10^6^ viable EAT cells and treated with GA or CA at a dose of 40 mg/kg and 80 mg/kg *ip* in exponential tumor growth phase on days 5, 7, 9, and 11. The results are expressed as the mean value of each experimental group ± SE of the mean (*n* = 11). ^b^ T/C% = T/C × 100; T, mean survival days of treated group; C, mean survival days of control group treated with saline. ^c^ ILS% (increased life span %) = (T–C)/C × 100. ^d^ LTS%, long-term survivors: mice surviving more than 67 days after the *ip* inoculation of EAT cells. Abbreviations: EAT, Ehrlich ascites tumor; GA, gallic acid; CA, caffeic acid.

**Table 3:** Effect of GA and CA on the nitric oxide (NO) and arginase 1 (Arg1) activity in the supernatant of the spleen, ascites macrophages, and ascites fluid of mice bearing EAT.

Experimental Group^a^	NO Levels in Supernatant of Macrophages or Ascites Fluid (SV ± SE)			Arg 1 Levels in Supernatant of Macrophages or Ascites Fluid (SV ± SE)		
	Spleen Macrophages	Ascites Macrophages	Ascites Fluid	Spleen Macrophages	Ascites Macrophages	Ascites Fluid
Control	15.87 ± 0.91	10.16 ± 0.98	20.27 ± 0.77	10.64 ± 0.67	11.50 ± 0.95	161.28 ± 6.28
GA 40 mg/kg	12.39 ± 1.47	19.62 ± 0.86 *	10.57 ± 0.55 ***	7.89 ± 0.89 *	9.65 ± 0.86	85.83 ± 0.54 ***
GA 80 mg/kg	11.98 ± 0.94	23.71 ± 1.04 **	15.86 ± 0.94	8.56 ± 0.04 **	8.47 ± 0.77 *	114.57 ± 4.34 *
CA 40 mg/kg	10.69 ± 0.95 *	10.16 ± 1.12 ^♦^	14.71 ± 0.39 *	7.80 ± 1.11	8.38 ± 0.50 **	129.96 ± 2.49 ^◊^
CA 80 mg/kg	10.78 ± 1.04 *	12.02 ± 1.29	18.68 ± 0.66 ^♦♦^	6.54 ± 0.15 **	8.07 ± 0.07 ***^♦^	110.10 ± 4.80 *

^a^ Mice were injected intraperitoneally (*ip*) with 2.5 × 10^6^ viable EAT cells and treated with GA or CA at a dose of 40 mg/kg and 80 mg/kg *ip* in exponential tumor growth phase on day 5, 7, 9, 11. Mice were sacrificed on the 13th day and the supernatant of the spleen, ascitic macrophages, and ascites fluid were collected and the NO and Arg 1 levels were estimated by ELISA. The results are expressed as the mean value of each experimental group ± SE of the mean of three different observations; * *p* < 0.05,** *p* < 0.01, *** *p* < 0.001 as compared to the control group treated with saline; ^♦^ *p* < 0.05, ^♦♦^ *p* < 0.01 as compared to GA-40. Abbrevations: EAT, Ehrlich ascites tumor; GA, gallic acid; CA, caffeic acid.

**Table 4:** Effect of GA and CA on oxidative stress markers in the ascites fluid of mice bearing EAT.

Experimental Group ^a^	MDA (nmol/mg Proteins)	GSH (µM/mL)	CAT Activity (U/mg Proteins)	SOD Activity (U/mg Proteins)
Control	12.41 ± 1.38	19.42 ± 2.30	0.26 ± 0.07	2.55 ± 0.17
GA 40 mg/kg	8.18 ± 1.80	19.06 ± 1.32	0.74 ± 0.07 *	5.82 ± 0.73
GA 80 mg/kg	9.25 ± 0.82	9.82 ± 0.72	0.25 ± 0.03	5.83 ± 0.86
CA 40 mg/kg	5.95 ± 0.51 *	13.42 ± 1.53	0.31 ± 0.04	3.01 ± 0.27
CA 80 mg/kg	11.10 ± 1.04	9.28 ± 1.64	0.44 ± 0.11	2.45 ± 0.35

^a^ Mice were injected intraperitoneally (*ip*) with 2.5 × 10^6^ viable EAT cells and treated with GA or CA at a dose of 40 mg/kg and 80 mg/kg *ip* in exponential tumor growth phase on days 5, 7, 9, and 11. The results are expressed as the mean value of each experimental group ± SE (*n* = 7). Mice were sacrificed on the 13th day, ascites was collected immediately after anesthesia, and oxidative stress markers (MDA, GSH, CAT, SOD) were measured as described in Materials and Methods. Data are reported as the mean ± SE of the mean of three different observations; * *p* < 0.05, as compared to the control group treated with saline. Abbreviation: EAT, Ehrlich ascites tumor; GA, gallic acid; CA, caffeic acid.

## Supplementary Materials

The following are available online. Figure S1: Effect of GA and CA on body weight change (%) of mice bearing EAT.
