# Supplementary material for: Natural Phenolic Acid, Product of the Honey Bee, for the Control of Oxidative Stress, Peritoneal Angiogenesis, and Tumor Growth in Mice
Source: Molecules. 2020 Nov 27;25(23):5583. doi: 10.3390/molecules25235583 (PMC7730286; doi:10.3390/molecules25235583)
Supplement: Supplementary file 1 [file molecules-25-05583-s001.pdf]

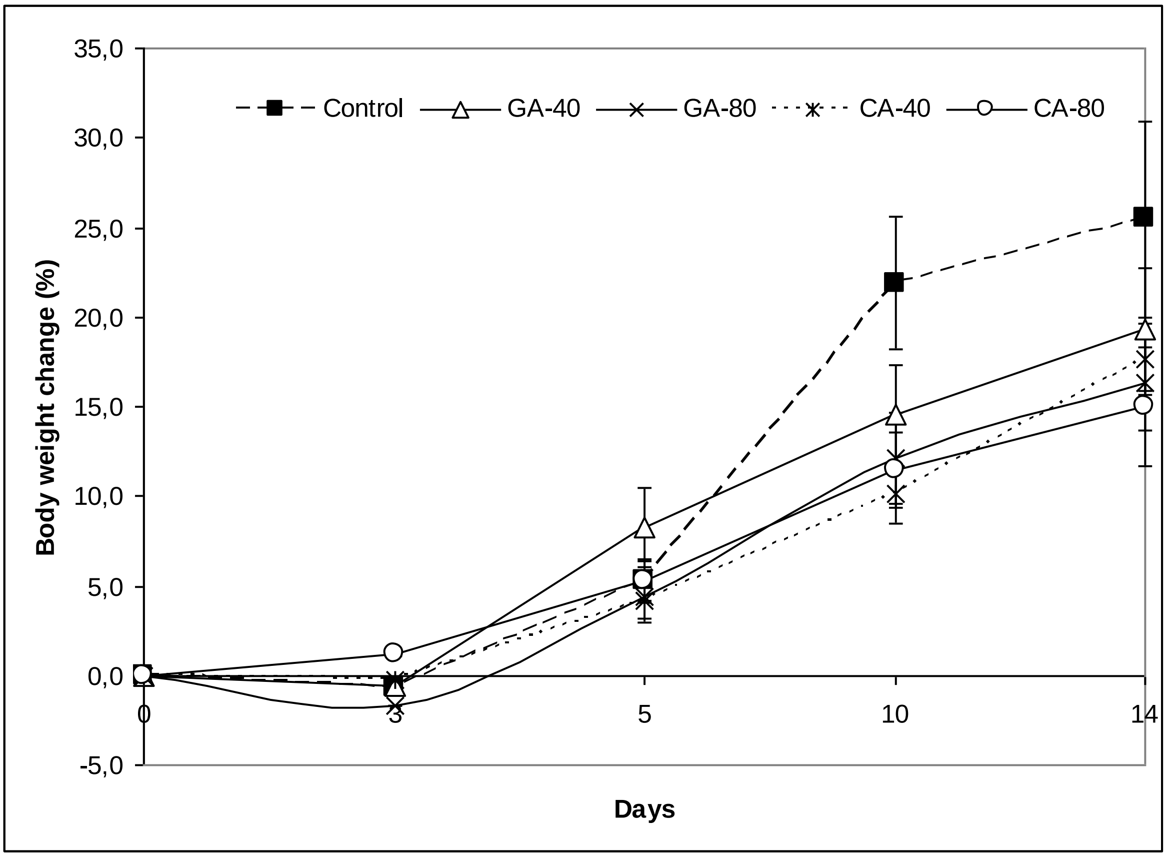

Figure S1. Effect of GA and CA on body weight change (%) of mice bearing EAT. Mice were injected intraperitoneally (*ip*) with 2.5x10^6 viable EAT cells and treated with GA or CA at a dose of 40 and 80 mg/kg *ip* in exponential tumor growth phase on days 5, 7, 9, and 11. The weight of the animals was recorded every five days from the day of inoculation (day 0) to sacrifice (day 13th). Percentage change in weight was calculated for individual animals as follows: Percentage change in weight = Final weight - Initial weight × 100/Final weight. The results are expressed as the mean value of each experimental group (n = 7). Abbreviations: EAT, Ehrlich ascites tumor; GA, gallic acid; CA, caffeic acid.
